# Prospects and Perspectives of Health Impact Assessment: A Systematic Review of the Peer-Reviewed Literature From June 2007 to January 2023

**DOI:** 10.3389/phrs.2024.1606649

**Published:** 2024-04-16

**Authors:** Nina Lamprecht, Tobias E. Erlanger, Jürg Utzinger, Mirko S. Winkler

**Affiliations:** ^1^ Swiss Tropical and Public Health Institute (Swiss TPH), Allschwil, Switzerland; ^2^ Swiss Federal Institute of Technology (ETH Zürich), Zürich, Switzerland; ^3^ University of Basel, Basel, Switzerland

**Keywords:** health impact assessment, health inequity, research-driven HIA, step-by-step HIA, systematic review

## Abstract

**Objectives:**

In 2008, an analysis investigating health impact assessment (HIA) practice found that only 6% of HIA-related peer-reviewed publications had a focus on low- and medium-developed countries, whereas 94% were conducted in countries with a high or very high development state. We aimed to update and deepen these observations.

**Methods:**

We conducted a systematic review, searching PubMed and Web of Science for HIA-related papers published in the scientific literature from June 2007 to January 2023. Only applied HIA and papers with HIA as a subject were included.

**Results:**

The search yielded 3,036 publications and the final selection consisted of 1,019 publications. The annual number of total publications increased considerably over the past 15 years. Whereas research-driven HIA (*n* = 460) showed a steep increase, step-by-step HIA (*n* = 71) did not show a clear trend.

**Conclusion:**

The gap between the number of HIA-related peer-reviewed publications focusing on low/medium and high/very high developed countries has diminished from 6/94 to 11/89. There is a growing tendency to apply the terminology HIA for health impact modelling studies and quantitative health risk assessments.

## Introduction

Health impact assessment (HIA) is a process that “systematically judges the potential, and sometimes unintended, effects of a project, program, plan, policy, or strategy [hereafter referred to as development initiative] on the health of a population and the distribution of those effects within the population. HIA generates evidence for appropriate actions to avoid or mitigate health risks and promote health opportunities. HIA guides the establishment of a framework for monitoring and evaluating changes in health as part of performance management and sustainable development” [1]. Although HIA aspires to provide evidence-based decision-support to development initiatives worldwide, a systematic literature review published by Erlanger et al. in 2008 found that only 6% of published HIA-related publications, referenced by the Web of Science and PubMed, had an explicit focus on low- and medium-developed countries, whereas the large majority, 94%, were conducted in countries with a high or very high development state. These observations pointed out a lack of HIA in large parts of the world and are even more concerning because low- and middle-income countries are often disproportionally affected by adverse health impacts due to anthropogenically amplified environmental changes [2].

Over the past 15 years much has happened in the field of HIA [3]. Several countries, including Brazil, Estonia, India, Ireland, Italy, Mexico, Norway, Slovakia, South Africa, Spain and Vietnam have defined guidelines or legal frameworks for promoting and regulating HIA practice [4]. Moreover, health has been specified to be a mandatory environmental factor of assessment in environmental impact assessment (EIA) by the European Union [5]. The International Council on Minerals and Metals (ICMM) published a good practice guidance on HIA in 2010 [6] and the International Petroleum Industry Environmental Conservation Association (IPIECA) issued an updated version of their HIA guidance in 2016 [7]. Major development financing institutions have issued HIA guidance. For example, the International Finance Corporation (IFC) published an introduction to HIA in 2009 [8] and the Asian Development Bank (ADB) issued an HIA source book in 2018 [9]. Finally, the International Association of Impact Assessment (IAIA) provided an updated version of their HIA international best practice principles in 2021 [1].

The surge in HIA guidance documents was accompanied by diversification in HIA practice. On the one hand, incorporation of health in other forms of impact assessments [e.g., EIA, environmental and social impact assessment (ESIA), environmental, social and health impact assessment (ESHIA), and strategic environmental assessment (SEA)] has become common practice [3]. On the other hand, a division in the overall methodological approach to HIA has become evident. The classical “step-by-step HIA,” which aims to support decision-making processes and involves stakeholder participation, is usually composed of the following main steps: 1) screening; 2) scoping; 3) impact assessment; 4) reporting; and 5) implementation and monitoring [1]. And “research-driven HIA,” which, in most cases, are not directly tied to decision-making processes of specific development initiatives, but instead are primarily driven by research interest [10]. For example, the health impacts of scenarios with less air pollution are compared to the health impacts of measured air pollution [11, 12]. Research-driven HIA is often limited to quantifying or modeling health impacts with ready-to-use tools such as AirQ or Dynamo-HIA [13, 14]. Hence, research-driven HIA usually focus on a few health outcomes, while step-by-step HIA generally apply a more holistic perspective on potentially affected health determinants and outcomes and adheres to more standardised frameworks [1]. Just as the two types of HIA differ in the overall methodological approach, there is little overlap in authorship, as reported in a recent study [15].

In view of the dynamic field of HIA, this paper aims to provide an overview of key developments in the scientific literature related to HIA over the past 15 years and to determine whether the 6/94 gap articulated in 2008 remained or had been improved or worsened. Emphasis is placed on the geographical focus of HIA-related papers published in the peer-reviewed literature since June 2007.

## Methods

We adhere to the method employed by Erlanger et al. (2008) and systematically reviewed the literature that has been published over the past 15 years. The main difference is that we only included peer-reviewed publications, while Erlanger et al. (2008) looked at various publication types (i.e., articles and reviews, meeting abstracts, editorials, letters and comments, books, book reviews, and HIA reports). The calculated gap in the Erlanger et al. paper is consistent for articles and reviews exclusively compared to the total gap—this is why we assume that the comparability between the studies is still given. As another change in the methods, we stratified HIA-related publications into specific categories (e.g., research-driven HIA and step-by-step HIA), whereas Erlanger et al. (2008) only differentiated between applied HIA and publications that featured HIA as a topic.

### Screening of the Peer-Reviewed Literature

A systematic literature review was conducted following the guidelines in the PRISMA statement [16]. Peer-reviewed publications concerning HIA were searched, applying the developed search strategy in PubMed and Web of Science. In both databases, records published from 1 June 2007 to 7 January 2023 were included. In terms of publication type, we explicitly searched for reviews and articles. The English term “health impact assessment” was used to narrow the search on the topic of HIA. The exact search terminology is summarised in Table 1.

**TABLE 1:** Search terminology used for each database (systematic review, global, 2007–2023).

Database	Query
PubMed	(((“health impact assessment”) AND ((“2007/06/01” [Date—Publication]: “3,000” [Date - Publication]))) AND ((“review” [Publication Type]) OR (“systematic review” [Publication Type]) OR (“journal article” [Publication Type]))
Web of Science	((ALL=(“health impact assessment”)) AND DOP=(2007-06-01/2023-01-07)) AND DT=(Review OR Article)

All records were exported to Rayyan (Rayyan Systems, Inc., Massachusetts, United States) for removing duplicates and screening titles and abstracts. During the screening, the publications were checked for inclusion and exclusion. For inclusion of a publication, HIA had to be either its methodological framework (“applied HIA”) or its focus (“HIA as a topic”). Applied studies that did not include “health impact assessment” in the title, abstract, or full text were excluded, just like papers that could not be classified according to their abstracts in case there was no full text available. Lastly, if the publication type was a conference paper, a dissertation, or a meeting abstract, it was excluded. Papers were categorized in “applied HIA” versus “HIA as a topic.”

In a second step, the full texts of the identified publications were screened and specific characteristics were extracted to an Excel spreadsheet. The extracted information included the country to which the first author was affiliated, the focus country of the publication, and whether the HIA presented belonged to the research-driven or step-by-step HIA category. In the latter case, it was specified whether the paper focused on evaluating a program, policy, project, or plan, or whether the paper was of a general nature.

### Data Analysis

Data were analyzed using Python 3.10 in PyCharm and JupyterHub. The following packages were used: pandas (version 1.3.5), geopandas (0.10.2), pycountry (22.3.5), mapclassify (2.5.0), and matplotlib (3.5.5). The international standard for country codes (ISO 3166) was used to create a world map. The PRISMA flow chart was made using lucidchart (Lucid Software Inc., Utah, United States).

### HDI Classification

Countries were classified as “low/medium development state” (HDI <0.7) or “high/very high development state” (HDI ≥0.7) according to the Human Development Index (HDI) [17]. The respective HDI was chosen from the year of publication. Publications with no specific focus country were labelled as general and publications with multiple focus countries were classified as “supranational”. While general publications were all labelled as having an “unclassifiable” development state, some supranational studies were assigned to a low/medium or high/very high development state, depending on the countries included.

For comparing the proportion of publications from countries with low/medium development state versus high/very high development state, publications that could not clearly be assigned to a development state were omitted.

### Ethical Considerations

Our systematic review does not involve human subjects, human tissues, or animal participants. Therefore, the approval of an ethics committee was not required.

## Results

### PRISMA Flow Chart

**FIGURE 1:**
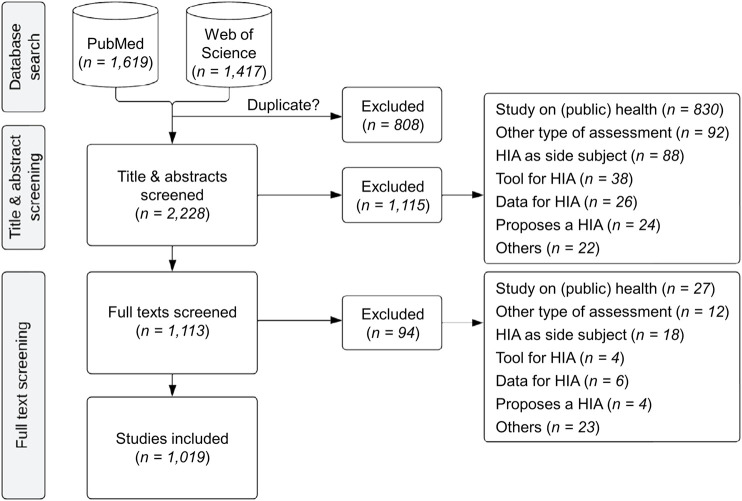
PRISMA flow chart showing the details of the screening process (systematic review, global, 2007–2023).

The total number of raw hits from PubMed was 1,619 and from Web of Science 1,417. Of those, 808 records were excluded as they were duplicates. From the 2,228 remaining records 1,115 publications were excluded after screening of titles and abstracts, mainly because the publications were only focusing on health or public health (*n* = 830) without a specific focus on HIA. Finally, as part of the full text screening of the 1,113 remaining publications, another 94 were excluded, leaving 1,019 for final analysis. A detailed overview on the screening process is shown in a PRISMA flow chart in Figure 1.

### Study Characteristics

Of the 1,019 publications included, 531 (52%) were applied HIA, whereas 488 (48%) had HIA as a topic. The latter contains, among others, methodological frameworks, evaluations of HIA practices in different countries, and opinions on the use of HIA. Only 71 (13%) of all applied HIA followed a classical step-by-step approach. The remaining 460 (87%), labelled as “research-driven HIA,” primarily contained quantitative HIA focusing mainly on the impact of air pollution on health. Also included in the research-driven category were HIA with a comprehensive view on health, taking into account several health determinants and health outcomes, but which did not follow a step-by-step approach. The step-by-step HIA were subdivided into HIA on projects or plans (*n* = 31; 44%), HIA on policies (*n* = 20; 28%), HIA on programs (*n* = 3; 4%), and general ones (*n* = 17; 24%).

### Geographical Distribution of HIA

**FIGURE 2:**
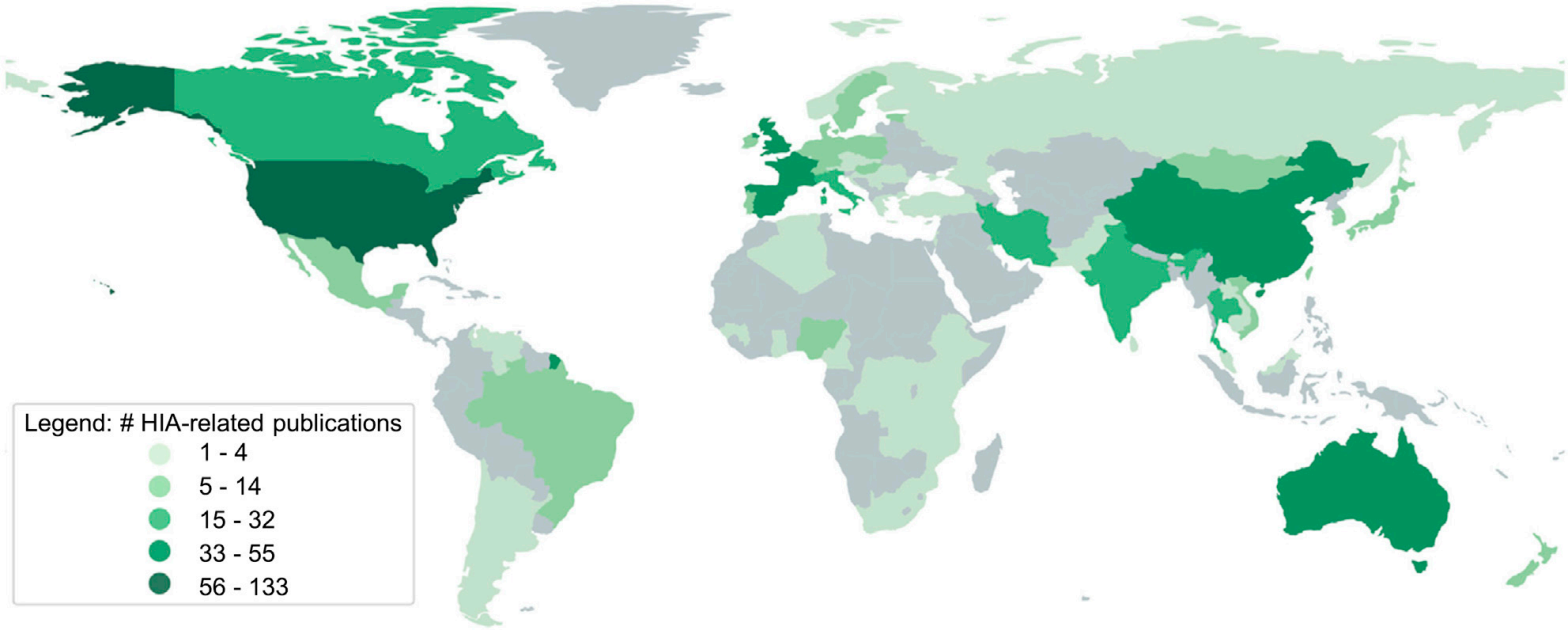
Numbers of health impact assessment (HIA)-related publications per focus country identified (systematic review, global, 2007–2023).

The largest proportion of the publications had no focus country, and thus were labelled as “general” (*n* = 190). An overview of HIA-publications by country is depicted in Figure 2. The complete list for each country and all study types is available in the Supplementary Appendix Table SA2.1.

Overall, the largest proportion of studies focused on the United States (*n* = 133). Another large proportion of 124 studies focused on multiple countries and were therefore labelled as “supranational”. Fifty-five articles focused exclusively on the United Kingdom, closely followed by publications focusing on the People’s Republic of China (*n* = 48), Spain (*n* = 41), Australia (*n* = 40), France (*n* = 39), Iran (*n* = 32), Italy (*n* = 29), Canada (*n* = 24), Thailand (*n* = 19), India (*n* = 19), and Brazil (*n* = 14). The remaining publications had their focus on 67 countries, each of which had HIA-publication-counts ranging between 1 and 11.

Papers presenting research-driven HIA were assigned to 65 countries. Most were from the United States (*n* = 57), followed by supranational (*n* = 53), People’s Republic of China (*n* = 43), United Kingdom (*n* = 26), Spain (*n* = 23), Iran (*n* = 22), France (*n* = 20), and those with a general focus (*n* = 21). Papers presenting step-by-step HIA were only found for 23 countries, 18 of which were from the United States. Twelve HIA were conducted in Australia, seven in the United Kingdom and five in Spain. In the remaining 19 countries, three or fewer step-by-step HIA were conducted.

Publications with HIA as a topic focused on 45 different countries, while most were general (*n* = 169) or had a supranational focus (*n* = 68). A total of 58 topic-publications focused on the United States, 22 on the United Kingdom, 21 on Canada, 18 on France, 16 on Australia, 13 on Spain, 12 on Italy, 10 on Thailand, and nine on Iran. For a graphical overview on the global distribution of the different study types, see Supplementary Appendix SA1.

### Differences Across Development States

Grouping the countries according to their development state provides a clearer picture: from all HIA-related publications; 221 could neither be assigned to “low/medium development state” nor “high/very high development state,” and hence, they were considered unclassifiable. Overall, 712 of the HIA-related publications had one or multiple high/very high developed countries as a focus, while 86 had a focus on one or multiple low/medium developed countries. Considering the 798 classifiable studies, 11% of HIA-related publications focused on low/medium developed countries, while 89% focused on high/very high developed countries.

The same proportion (11% low/medium and 89% high/very high) resulted when considering research-driven HIA: 389 publications focused on high/very high and 49 on low/medium developed countries, while 33 could not be classified. Likewise, 11% (*n* = 33) of papers that had HIA as a topic focused on low/medium developed countries, whereas 89% (*n* = 268) focused on high/very high developed countries; omitting the 186 papers which were not classifiable.

**FIGURE 3:**
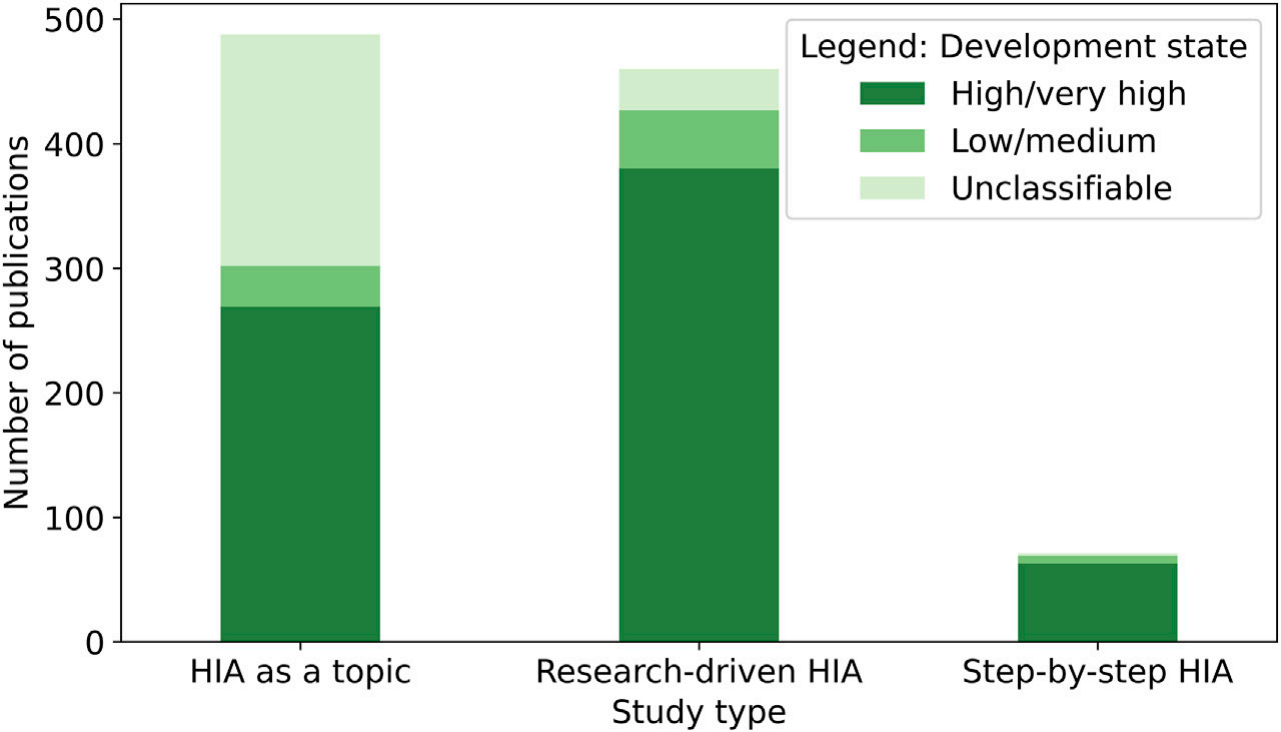
Numbers of health impact assessment (HIA)-related publications identified, stratified by study type (systematic review, global, 2007–2023). Note: studies were categorized by the Human Development Index (HDI) of their focus countries. Papers with multiple focus countries or with a general focus are labelled as “unclassifiable”.

The proportions of step-by-step HIA were even more imbalanced: 55 (92%) had a focus on high/very high developed countries, whereas only five (8%) had a focus on low/medium developed countries. Two step-by-step HIA were unclassifiable. The number of publications of the different study types grouped by their development state classifications are depicted in Figure 3.

### Country Affiliation of First Authors

While the HIA-related scientific publications focused on 80 different countries, the first authors of those were affiliated with 62 different countries. With a total of 181 first authors affiliated to the United States, this was by far the leading country, followed by the United Kingdom (*n* = 117). Further countries from which many first authors originate are Australia (*n* = 77), Spain (*n* = 76), Canada (*n* = 52), France (*n* = 52), People’s Republic of China (*n* = 48), Italy (*n* = 47), Switzerland (*n* = 38), Netherlands (*n* = 32), Iran (*n* = 31), Thailand (*n* = 21), India and Germany (both *n* = 18). Of all publications focusing on a low/medium developed country, 56 of their first authors were affiliated with a high/very high developed country, whereas only 31 were affiliated with low/medium developed countries. Publications focusing on high/very high countries were exclusively written by first authors affiliated with high/very high countries.

Narrowing the focus to the different types of studies, research-driven HIA were written by first authors affiliated with institutions in 53 different countries. Seventy-two first authors came from the United States, 45 from the United Kingdom, 42 from Spain, 41 from the People’s Republic of China, 26 from France, 22 from Iran, 20 from Italy, and 18 were written by first authors from Australia. Other countries had 12 or less first authors affiliated with a specific country and contributing to research-driven HIA. First authors in step-by-step HIA were affiliated with 19 different countries: United States (*n* = 17), Australia (*n* = 13), United Kingdom and Spain (both *n* = 6). HIA-topic publications had first authors of 39 different countries: United States (*n* = 92), United Kingdom (*n* = 66), Canada (*n* = 47), Australia (*n* = 46), Spain (*n* = 28), Switzerland (*n* = 27), France and Italy (both *n* = 25), and the Netherlands (*n* = 19).

### Temporal Trends

**FIGURE 4:**
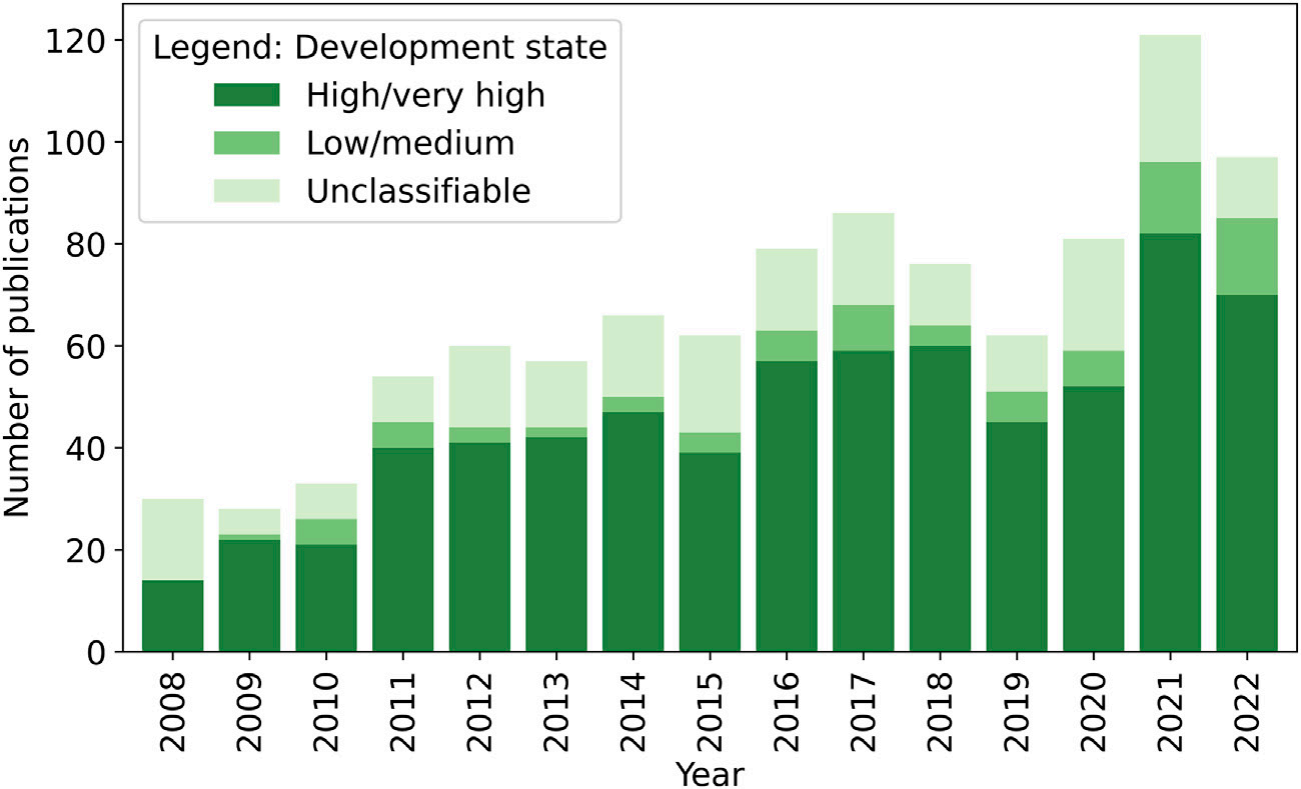
Number of health impact assessment (HIA)-related publications identified, categorized by the Human Development Index (HDI) of their focus countries (systematic review, global, 2008–2023). Note: papers with multiple focus countries or with a general focus were labelled as “unclassifiable”.

As the observed periods in 2007 and 2023 only cover months or even days, we exclusively compare the complete years and show only those in the figures. From 2008 until 2022, an increasing trend in the annual numbers of HIA-related publications can be observed (Figure 4). The absolute increase was most prominent in studies focusing on high/very high developed countries (2008: *n* = 14, 2021: *n* = 82). Also, studies with a focus on low/medium developed countries showed an increase (2008: *n* = 0, 2021: *n* = 14).

**FIGURE 5:**
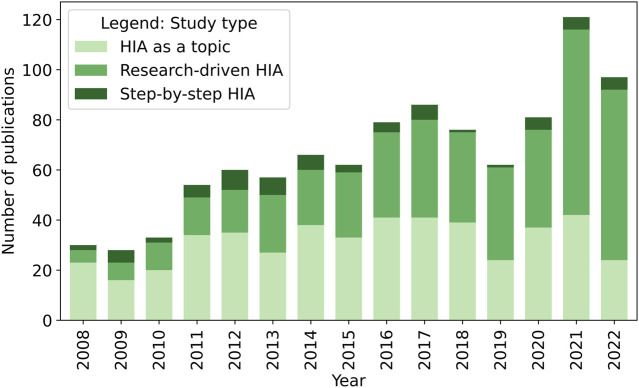
Number of health impact assessment (HIA)-related publications identified, stratified by study type (systematic review, global, 2008–2023).

An upward trend is also visible when looking at the different types of studies (Figure 5). The highest increase of HIA-related papers is owed to research-driven HIA, rising from 5 in 2008 to 70 in 2022. For HIA as a topic, a slight upward trend was observed from 2008 (*n* = 23) until 2017 (*n* = 41), stabilising or even slightly decreasing until 2022. For step-by-step HIA, no distinct trend could be observed.

## Discussion

We systematically searched two of the most widely used electronic databases in biomedical research (i.e., PubMed and Web of Science) for HIA-related peer-reviewed articles published from mid-2007 until early 2023. Publications were sorted by their focus countries, the development state of the focus countries, the study type (i.e., research-driven HIA, step-by-step HIA, and HIA as a topic), and affiliation of first authors with the aim to unveil current trends in the HIA-related scientific literature and, thus, to investigate whether the 6/94 gap in HIA reported in 2008 prevailed [18]. While Erlanger et al. identified 237 HIA-related publications between 1976 and May 2007, our search yielded 1,019 peer-reviewed papers published between June 2007 and January 2023. The observed acceleration in the number of publications was paralleled by narrowing the gap between low/medium and high/very high HDI countries; the gap declined from 6/94 to 11/89. The increasing number of total papers is primarily attributable to a steep rise in modeling and risk assessment studies (classified as “research-driven HIA”), whereas the number of peer-reviewed papers presenting step-by-step HIA remained relatively stable.

### Change in the Geographical Distribution of HIA-Related Publications

Not only has the number of published HIA-related papers increased considerably, but also their geographical dispersion has changed. While Erlanger et al. (2008) identified HIA-related publications focusing on 23 countries, the number of 80 focus countries found in the current analysis shows a much wider distribution. However, this wide spread of HIA-related peer-reviewed publications must be put into perspective, as it is primarily driven by research-driven HIA, which themselves are a wide array of many different types of studies, for example, quantitative HIA following various methods and qualitative HIA that did not follow a step-by-step method. Most of the included step-by-step HIA focused on high/very high developed countries. Hence, recent efforts of international institutions, including ADB, the African Development Bank, the East and Southern African Management Institute, ICMM, IFC, IPIECA, and the World Bank seem to have had little effect in the number of peer-reviewed papers in the field of HIA deriving from low/medium developed countries [19]. At the same time, our systematic review of the peer-reviewed literature does not allow to draw conclusions on trends in global HIA practice, which is further addressed in the limitations section.

### Step-by-Step HIA

Being clearly outnumbered by research-driven publications and publications with HIA as a topic, the number of published step-by-step HIA does not show a distinct trend. This is a surprising finding as step-by-step HIA are the central topic of a large proportion of the HIA-topic papers. A potential explanation for the observed discrepancy could be that step-by-step HIA are often not published in the peer-reviewed literature, perhaps explained by a lack of interest, or monetary or time restrictions [18, 20]. Finally, a substantial number of HIA, especially if commissioned by the private sector, might be kept confidential, as shown in a recent study by Dietler et al. [21]. Hence, a search including grey literature and HIA specific databases would be needed to produce a comprehensive overview of step-by-step HIA practice.

### Research-Driven HIA

While only a few research-driven HIA were published in 2007 and 2008, they formed the large majority of studies included in our search. Such a rapid evolution of research-driven HIA, most of which are quantitative HIA, is encouraging since quantification in HIA was found to be largely absent until 2004 [21, 22]. Several developments might have contributed to the observed trend. Firstly, it seems that over the past decade, the term “health impact assessment” has become a term that is used too quickly. Indeed, while screening the abstracts of identified papers, we observed that many records featured “health impact assessment” as a keyword, although the actual work had little to do with HIA. Hence, it seems necessary to clarify within the fields of public health and epidemiology that the original purpose of HIA is to make recommendations from a health perspective to improve a proposed project, program or policy. Studies that are, for example, primarily concerned with impacts that have occurred because of the implementation of a project, program or policy should instead be referred to as “impact evaluations” [23]. Similarly, one could argue that risk assessment or modeling studies that are not directly tied to decision-making processes, should avoid the terminology HIA and remain specific in the labelling of the research conducted. Secondly, research-driven HIAs are primarily conducted by the scientific community, which aspires to publish their work in the peer-reviewed literature, explaining the higher publication number compared to step-by-step HIA. Finally, the increasing trend in publications presenting research-driven HIA might also simply reflect an emerging and productive field of research. This is a positive development from a public health perspective as the quantification of health impacts of global challenges such as climate change [10], urbanization [24], and industrialization [25, 26] are essential to promote sustainability-oriented decision-making. However, since low- and medium-developed countries are expected to be particularly affected by adverse effects of climate change and urbanization, it seems essential to promote research-driven HIA oriented toward these countries.

### Limitations

This systematic review deepens the understanding of recent trends in the peer-reviewed literature in the field of HIA. However, the picture drawn by the current study might distort the reality for several reasons, particularly with regard to the very low numbers of identified step-by-step HIA—71 over the period of 15 years. Hence, our review cannot draw any conclusions on global trends in HIA practice since step-by-step HIA are often not published in the peer-reviewed literature. This might be because of monetary or time restrictions [18] or their proximity to industry and government rather than the publication-focused academic community. Thus, in order to observe trends in global HIA practice, grey literature or HIA-specific databases would need to be searched, which is beyond the scope of the present paper. Furthermore, our search of the peer-reviewed literature may be affected by a publication bias as, compared to high income countries, HIA investigators in low-income countries may have fewer resources or incentives to publish in the peer-reviewed literature. Finally, we acknowledge that our systematic review could have introduced a language bias: since HIA are often integrated into national or subnational processes, they might get reported in national languages [27]. The current study only searched for HIA in English, using the term “health impact assessment.” Hence, the observed dominance in focus countries with English as one of their spoken languages like the United States, United Kingdom, Canada, and Australia could be partly explained, while HIA from other regions are most likely underrepresented.

## Conclusion

The objective of this paper was to update the observed 6/94 gap in HIA research reported in 2008 and to examine trends in the scientific literature in the field of HIA over the past 15 years. Overall, 1,019 papers were included, underlining the increasing number of HIA-related peer-reviewed publications. They did not just increase in number—also the diversity of focus countries grew from 23 (from 1976 to May 2007) [18] to 80 (from June 2007 to January 2023 in our study). Although the scientific literature in the HIA field is still dominated by the English-speaking world and some European countries, more publications focusing on parts of Asia, Latin America, and some few on Africa were observed. However, the publication of step-by-step HIA in the scientific literature did neither seem to have increased nor decreased over the past 15 years. The observed increase in HIA-related publications is primarily attributed to the significant growth of research-driven HIA from 2007 onwards. The absolute number of HIA-related peer-reviewed publications increased for high/very high as well as for low/medium developed countries. Hence, the gap in HIA-related publications in the scientific literature narrowed from 6/94 to 11/89 over the past 15 years, which hints at a growing number of academics from low- and medium-developed countries who are active in the field of HIA.

## Abbreviations

ADB, Asian Development Bank; DALY, disability-adjusted life year; EIA, environmental impact assessment; ESHIA, environmental, social and health impact assessment; ESIA, environmental and social impact assessment; HDI, Human Development Index; HIA, health impact assessment; IAIA, International Association for Impact Assessment; ICMM, International Council on Minerals and Metals; IFC, International Finance Corporation; SDC, Swiss Agency for Development and Cooperation; SEA, strategic environmental assessment; SNSF, Swiss National Science Foundation; WHO, World Health Organization.

## References

[1] Winkler MS Viliani F Knoblauch AM Cave B Divall M Ramesh G Health Impact Assessment, International Best Practice Principles. Int Assoc Impact Assess (2021) 2021(5):551–5.

[2] Thondoo M Rojas-Rueda D Gupta J De Vries DH Nieuwenhuijsen MJ . Systematic Literature Review of Health Impact Assessments in Low and Middle-Income Countries. Int J Environ Res Public Health (2019) 16(11):2018. 10.3390/ijerph16112018 31174273 PMC6603924

[3] Winkler MS Furu P Viliani F Cave B Divall M Ramesh G Current Global Health Impact Assessment Practice. Int J Environ Res Public Health (2020) 17(9):2988. 10.3390/ijerph17092988 32344882 PMC7246701

[4] Thondoo M Gupta J . Health Impact Assessment Legislation in Developing Countries: A Path to Sustainable Development? Rev Eur Comp Int Environ L (2021) 30(1):107–17. 10.1111/reel.12347

[5] Cave B Claßen T Fischer-Bonde B Humboldt-Dachroeden S Martín-Olmedo P Mekel O Human Health: Ensuring a High Level of Protection. A Reference Paper on Addressing Human Health in Environmental Impact Assessment (2020). Available from: https://eupha.org/repository/sections/HIA/Human%20Health%20Ensuring%20Protection%20Main%20and%20Appendices.pdf (Accessed April 6, 2024).

[6] ICMM. Good Practice Guidance on Health Impact Assessment (2010). Available from: https://www.icmm.com/en-gb/guidance/health-safety/2010/guidance-hia (Accessed April 6, 2024).

[7] IPIECA. Health Impact Assessment A Guide for the Oil and Gas Industry (2016). Available from: https://www.ipieca.org/resources/health-impact-assessment-a-guide-for-the-oil-and-gas-industry (Accessed April 24, 2023).

[8] IFC. Introduction to Health Impact Assessment. Washington, DC: International Finance Corporation (2009). Available from: https://www.ifc.org/wps/wcm/connect/topics_ext_content/ifc_external_corporate_site/sustainability-at-ifc/publications/publications_handbook_healthimpactassessment__wci__1319578475704 (Accessed April 6, 2024).

[9] ADB. Health Impact Assessment A Good Practice Sourcebook (2018). Available from: https://www.adb.org/documents/health-impact-assessment-sourcebook (Accessed April 6, 2024).

[10] Ammann P Dietler D Winkler MS . Health Impact Assessment and Climate Change: A Scoping Review. J Clim Change Health (2021) 3:100045. 10.1016/j.joclim.2021.100045

[11] Safari Z Fouladi-Fard R Vahedian M Mahmoudian MH Rahbar A Fiore M . Health Impact Assessment and Evaluation of Economic Costs Attributed to PM2.5 Air Pollution Using BenMAP-CE. Int J Biometeorol (2022) 66(9):1891–902. 10.1007/s00484-022-02330-1 35852660 PMC9295116

[12] Kanhai G Fobil JN Nartey BA Spadaro JV Mudu P . Urban Municipal Solid Waste Management: Modeling Air Pollution Scenarios and Health Impacts in the Case of Accra, Ghana. Waste Manage (2021) 123:15–22. 10.1016/j.wasman.2021.01.005 33548745

[13] Kang E . Assessing Health Impacts of Pictorial Health Warning Labels on Cigarette Packs in Korea Using DYNAMO-HIA. J Prev Med Public Health (2017) 50(4):251–61. 10.3961/jpmph.17.032 28768403 PMC5541276

[14] Hadei M Hopke PK Nazari SSH Yarahmadi M Shahsavani A Alipour MR . Estimation of Mortality and Hospital Admissions Attributed to Criteria Air Pollutants in Tehran Metropolis, Iran (2013–2016). Aerosol Air Qual Res (2017) 17(10):2474–81. 10.4209/aaqr.2017.04.0128

[15] Kim J Haigh FA . HIA and EIA Are Different, But Maybe Not in the Way We Thought They Were: A Bibliometric Analysis. Int J Environ Res Public Health (2021) 18(17):9101. 10.3390/ijerph18179101 34501690 PMC8430742

[16] Moher D Liberati A Tetzlaff J Altman DG . Preferred Reporting Items for Systematic Reviews and Meta-Analyses: The PRISMA Statement. BMJ (Online) (2009) 339(7716):b2535–6. 10.1136/bmj.b2535 PMC2714657 19622551

[17] UNDP. Human Development Reports - All Composite Indices and Components Time Series (1990-2021) (2021). Available from: https://hdr.undp.org/sites/default/files/2021-22_HDR/HDR21-22_Composite_indices_complete_time_series.csv (Accessed April 6, 2024).

[18] Erlanger TE Krieger GR Singer BH Utzinger J . The 6/94 Gap in Health Impact Assessment. Environ Impact Assess Rev (2008) 28(4–5):349–58. 10.1016/j.eiar.2007.07.003

[19] Tetteh D Lengel L . The Urgent Need for Health Impact Assessment: Proposing a Transdisciplinary Approach to the E-Waste Crisis in Sub-Saharan Africa. Glob Health Promot (2017) 24:35–42. 10.1177/1757975916686926 28353403

[20] Pereira CAR Périssé ARS Knoblauch AM Utzinger J Hacon SS Winkler MS . Health Impact Assessment in Latin American Countries: Current Practice and Prospects. Environ Impact Assess Rev (2017) 65:175–85. 10.1016/j.eiar.2016.09.005

[21] Dietler D Lewinski R Azevedo S Engebretsen R Brugger F Utzinger J Inclusion of Health in Impact Assessment: A Review of Current Practice in Sub-Saharan Africa. Int J Environ Res Public Health (2020) 17(11):4155. 10.3390/ijerph17114155 32532108 PMC7312242

[22] Veerman JL Barendregt JJ Mackenbach JP . Quantitative Health Impact Assessment: Current Practice and Future Directions. J Epidemiol Community Health (2005) 59(5):361–70. 10.1136/jech.2004.026039 15831683 PMC1733071

[23] Kemm J . Health Impact Assessment: Past Achievement, Current Understanding, and Future Progress. Oxford: Oxford University Press (2012).

[24] Nieuwenhuijsen MJ Barrera-Gómez J Basagaña X Cirach M Daher C Pulido MF Study Protocol of the European Urban Burden of Disease Project: A Health Impact Assessment Study. BMJ Open (2022) 12(1):e054270. 10.1136/bmjopen-2021-054270 PMC8783806 35058262

[25] Farnham A Cossa DD Engebretsen R Leuenberger A Lyatuu I , Investigating Health Impacts of Natural Resource Extraction Projects in Burkina Faso, Ghana, Mozambique, and Tanzania: Protocol for a Mixed Methods Study. JMIR Res Protoc (2020) 9(4):e17138. 10.2196/17138 32266876 PMC7177430

[26] Winkler MS Adongo PB Binka F Brugger F Diagbouga S Macete E Health Impact Assessment for Promoting Sustainable Development: The HIA4SD Project. Impact Assess Project Appraisal (2020) 38(3):225–32. 10.1080/14615517.2019.1694783

[27] Winkler MS Krieger GR Divall MJ Cissé G Wielga M Singer BH Untapped Potential of Health Impact Assessment. Bull World Health Organ (2013) 91(4):298–305. 10.2471/BLT.12.112318 23599554 PMC3629454

