# Supplementary material for: Prospects and Perspectives of Health Impact Assessment: A Systematic Review of the Peer-Reviewed Literature From June 2007 to January 2023
Source: Public Health Rev. 2024 Apr 16;45:1606649. doi: 10.3389/phrs.2024.1606649 (PMC11059091; doi:10.3389/phrs.2024.1606649)
Supplement: Supplementary file 1 [file DataSheet1.docx]

**Appendix A: Supplementary material**


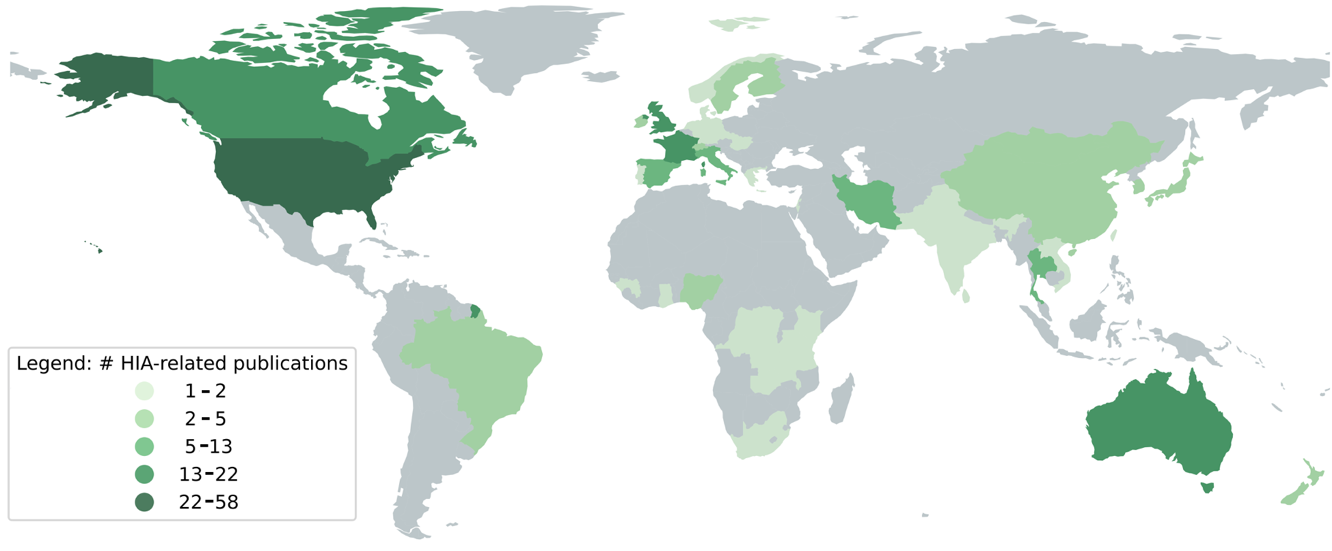
Appendix A1: Global distribution of the different study types.


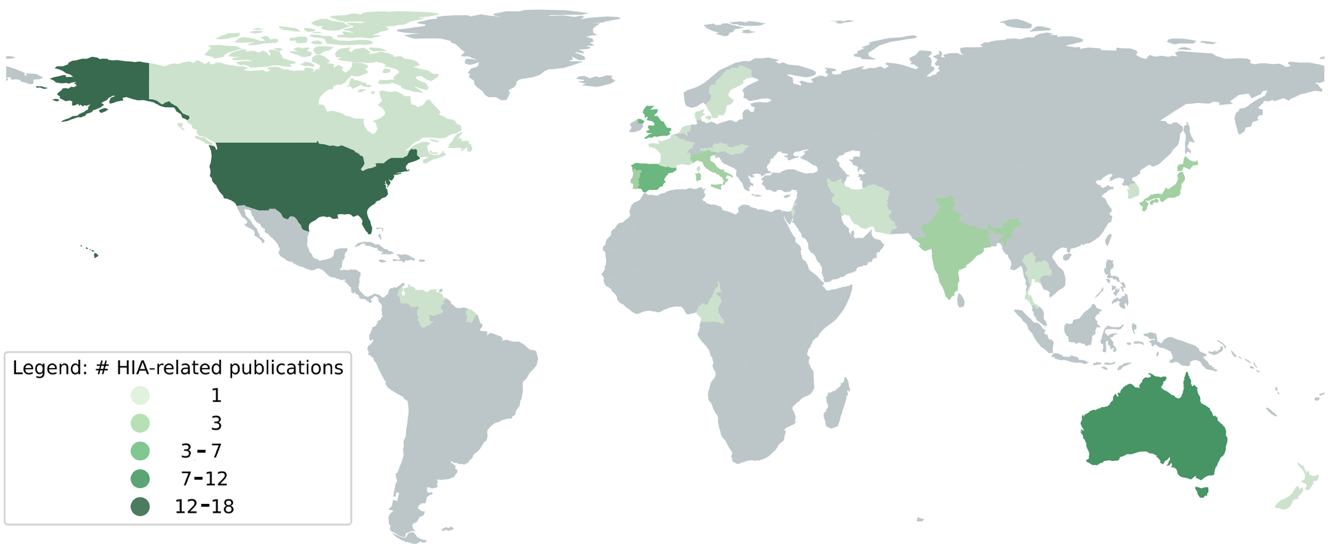
**Figure A1.1**: Global distribution of publications with HIA as a topic, published between June 2007 and January 2023 according to a systematic search on Web of Science and PubMed. Gray indicates that no HIA-related publication with the focus on the respective country was found.

**Figure A1.2**: Global distribution of step-by-step HIA published between June 2007 and January 2023 according to a systematic search on Web of Science and PubMed. Gray indicates that no HIA-related publication with the focus on the respective country was found.


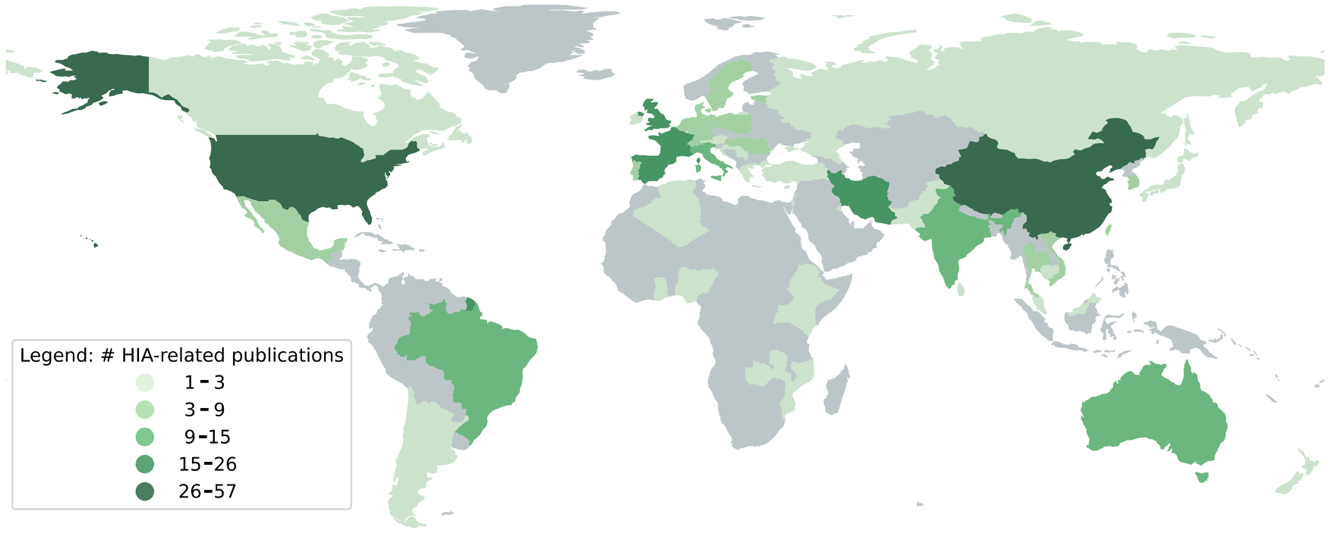
**Figure A1.3**: Global distribution of research-driven HIA published between June 2007 and January 2023 according to a systematic search on Web of Science and PubMed. Gray indicates that no HIA-related publication with the focus on the respective country was found.

Appendix A2: Publication counts.

**Table A2.1:** Number of HIA-related publications identified in this systematic review per country and per study type.

| **Development state** | **Focus country** | **Step-by-step** | **Research-driven** | **HIA as a topic** | **Total** |
| --- | --- | --- | --- | --- | --- |
| High / very high | **Algeria** | 0 | 1 | 0 | **1** |
|  | **Argentina** | 0 | 1 | 0 | **1** |
|  | **Australia** | 11 | 13 | 16 | **40** |
|  | **Austria** | 0 | 3 | 0 | **3** |
|  | **Belgium** | 0 | 6 | 0 | **6** |
|  | **Brazil** | 0 | 10 | 4 | **14** |
|  | **Canada** | 1 | 2 | 21 | **24** |
|  | **Chile** | 0 | 1 | 0 | **1** |
|  | **China** | 0 | 42 | 5 | **47** |
|  | **Croatia** | 0 | 1 | 0 | **1** |
|  | **Cyprus** | 0 | 1 | 0 | **1** |
|  | **Czechia** | 0 | 0 | 1 | **1** |
|  | **Denmark** | 0 | 7 | 2 | **9** |
|  | **Estonia** | 0 | 6 | 0 | **6** |
|  | **Finland** | 0 | 0 | 3 | **3** |
|  | **France** | 1 | 20 | 18 | **39** |
|  | **Germany** | 0 | 4 | 1 | **5** |
|  | **Greece** | 0 | 3 | 1 | **4** |
|  | **Hong Kong** | 0 | 1 | 0 | **1** |
|  | **Hungary** | 1 | 5 | 1 | **7** |
|  | **Iran** | 1 | 23 | 9 | **33** |
|  | **Ireland** | 0 | 1 | 4 | **5** |
|  | **Israel** | 1 | 1 | 1 | **3** |
|  | **Italy** | 2 | 15 | 12 | **29** |
|  | **Japan** | 2 | 3 | 3 | **8** |
|  | **Kuwait** | 0 | 1 | 0 | **1** |
|  | **Luxembourg** | 0 | 1 | 0 | **1** |
|  | **Malaysia** | 0 | 1 | 0 | **1** |
|  | **Mexico** | 0 | 6 | 0 | **6** |
|  | **Mongolia** | 0 | 1 | 4 | **5** |
|  | **Netherlands** | 1 | 5 | 2 | **8** |
|  | **New Zealand** | 1 | 1 | 3 | **5** |
|  | **Norway** | 0 | 0 | 1 | **1** |
|  | **Poland** | 0 | 9 | 0 | **9** |
|  | **Portugal** | 2 | 6 | 1 | **9** |
|  | **Puerto Rico** | 1 | 0 | 0 | **1** |
|  | **Romania** | 0 | 4 | 0 | **4** |
|  | **Russian Federation** | 0 | 1 | 0 | **1** |
|  | **Serbia** | 0 | 1 | 0 | **1** |
|  | **Slovakia** | 0 | 0 | 2 | **2** |
|  | **South Africa** | 0 | 1 | 0 | **1** |
|  | **South Korea** | 1 | 6 | 4 | **11** |
|  | **Spain** | 5 | 23 | 13 | **41** |
|  | **Sri Lanka** | 0 | 1 | 1 | **2** |
|  | **Sweden** | 1 | 6 | 3 | **10** |
|  | **Switzerland** | 0 | 5 | 3 | **8** |
|  | **Taiwan** | 0 | 4 | 1 | **5** |
|  | **Thailand** | 1 | 8 | 10 | **19** |
|  | **Turkey** | 0 | 3 | 0 | **3** |
|  | **United Kingdom** | 5 | 28 | 22 | **55** |
|  | **United States of America** | 16 | 59 | 58 | **133** |
|  | **supranational** | 1 | 38 | 38 | **77** |
| Low / medium | **Bhutan** | 0 | 0 | 1 | **1** |
|  | **Cambodia** | 0 | 1 | 0 | **1** |
|  | **Cameroon** | 1 | 0 | 0 | **1** |
|  | **China** | 0 | 1 | 0 | **1** |
|  | **Democratic Republic of the Congo** | 0 | 0 | 1 | **1** |
|  | **Ethiopia** | 0 | 3 | 0 | **3** |
|  | **Ghana** | 0 | 2 | 1 | **3** |
|  | **India** | 2 | 15 | 2 | **19** |
|  | **Kenya** | 0 | 1 | 1 | **2** |
|  | **Kiribati** | 0 | 1 | 0 | **1** |
|  | **Laos** | 0 | 0 | 1 | **1** |
|  | **Lesotho** | 0 | 0 | 1 | **1** |
|  | **Malawi** | 0 | 0 | 1 | **1** |
|  | **Mozambique** | 0 | 1 | 0 | **1** |
|  | **Myanmar** | 0 | 1 | 0 | **1** |
|  | **Nigeria** | 0 | 3 | 4 | **7** |
|  | **Pakistan** | 0 | 3 | 1 | **4** |
|  | **Republic of Guinea** | 0 | 0 | 2 | **2** |
|  | **Sierra Leone** | 0 | 1 | 0 | **1** |
|  | **Solomon Island** | 0 | 1 | 0 | **1** |
|  | **Tanzania** | 0 | 0 | 1 | **1** |
|  | **Uganda** | 0 | 2 | 0 | **2** |
|  | **Vanuatu** | 1 | 0 | 0 | **1** |
|  | **Venezuela** | 0 | 1 | 0 | **1** |
|  | **Vietnam** | 0 | 6 | 1 | **7** |
|  | **Zambia** | 0 | 3 | 1 | **4** |
|  | **supranational** | 1 | 3 | 14 | **18** |
| Unclassifiable | **general** | 1 | 21 | 169 | **191** |
|  | **supranational** | 1 | 12 | 16 | **29** |
